# Relationship Between Weight Status and Health-Related Quality of Life in School-age Children in China

**DOI:** 10.36469/jheor.2022.32414

**Published:** 2022-03-07

**Authors:** Mandana Zanganeh, Peymané Adab, Bai Li, Miranda Pallan, Wei J. Liu, Lin Rong, Wei Liu, James Martin, Kar K. Cheng, Emma Frew

**Affiliations:** 1 Centre for Health Economics, Warwick Medical School University of Warwick; 2 Institute of Applied Health Research University of Birmingham; 3 Centre for Exercise, Nutrition and Health Sciences, School for Policy Studies University of Bristol; 4 Institute of Applied Health Research, College of Medical and Dental Sciences University of Birmingham; 5 School Health Unit Centre for Disease Control and Prevention, Guangzhou, China

**Keywords:** weight status, overweight/obesity, health-related quality of life, children, China

## Abstract

**Background:** Some studies from high-income countries suggest that overweight and/or obesity in children are negatively associated with health-related quality of life (HRQOL). However, the relationship between weight status and HRQOL is not well established in China, where obesity trends follow a different pattern compared with high-income countries. The risk of obesity is greater in children from higher socioeconomic backgrounds and higher in boys compared with girls.

**Objective:** The aim of this study was to examine the relationship between weight status and HRQOL in children between 6 and 7 years old in this unique country context.

**Methods:** Baseline HRQOL and demographic data were collected from children recruited to the CHIRPY DRAGON obesity prevention trial in China. HRQOL was measured using the Chinese version of the Child Health Utility-9D (CHU-9D-CHN) and the Pediatric Quality of Life Inventory™ (PedsQL™) instruments. CHU-9D-CHN utility scores were generated using 2 scoring algorithms (UK and Chinese tariffs). Height and weight measures were taken at school by trained researchers using standardized methods, and BMI *z* scores were calculated using the World Health Organization 2007 growth charts. The relationship between HRQOL and weight status was examined using multivariable analyses, adjusting for age, gender, and socioeconomic status.

**Results:** Full data were available for 1539 children (mean age, 6 years). In both unadjusted and adjusted analyses, HRQOL, using both the CHU-9D-CHN and the PedsQL™, was marginally higher in children who were overweight or living with obesity compared with children with healthy weight, although this difference did not reach statistical significance. Separate analyses and models by gender showed that the relationship between weight status and HRQOL scores was similar in boys and girls.

**Conclusions:** Our results suggest no statistically significant difference in HRQOL between children with overweight/obesity compared with those with healthy weight. These results have implications for the methods of economic evaluation for obesity treatment and prevention interventions within this population cohort and country setting, as there appears to be no discernible consequences on children’s HRQOL from living with overweight and obesity.

## INTRODUCTION

Despite countless calls to action, tackling childhood obesity remains one of the most significant worldwide public health challenges of our time. Overweight and obesity are linked to serious physical health, emotional and social problems in both children and adults.^1–3^ For example, a growing body of evidence shows that obesity in childhood has a detrimental effect on health-related quality of life (HRQOL), as children living with severe obesity have reported HRQOL that is comparable with cancer.^4^ Adding to this complexity are indications that the relationship between weight status and HRQOL is sensitive to demographic factors such as age and socioeconomic status.^5–9^

However, in some cultures, such as within China, childhood obesity may not be perceived as ill health and may, in fact, be considered a sign of good health,^10–12^ so the relationship between HRQOL and obesity could be different. Also, obesity trends follow a different pattern in China compared with high-income countries (which are at a more advanced stage of the obesity epidemic), with the risk of obesity being greater in children from higher socioeconomic backgrounds and much greater in boys compared with girls.^13,14^

To date, very few studies have examined the relationship between weight status and HRQOL in children; the few that do exist are predominantly from Western or high-income countries.^4–9,15–23^ Some of these studies from high-income countries suggest that overweight and obesity in children is negatively associated with HRQOL,^4–8,19,20,22,23^ but the evidence is mixed. A better understanding of this relationship can help inform treatment decisions and is important in terms of evaluation of interventions to tackle obesity, particularly economic evaluations that rely on the assumption that reductions in overweight and obesity translate to improvements in HRQOL and are therefore captured within utility-based HRQOL measures used to construct quality-adjusted life years (QALYs).

This paper directly addresses this evidence gap by examining the relationship between weight status and HRQOL in children, using a utility-based measure of QOL (the Child Health Utility 9D [CHU-9D] instrument). The Pediatric Quality of Life Inventory™ (PedsQL™) instrument was used as a further assessment to ensure consistency of findings. Given the difference in childhood obesity risk across boys and girls in China, we also examined whether any relationship differed by gender.

## METHODS

### Data Source and Study Design

The analysis uses baseline data from 1539 out of 1640 children who took part in the CHIRPY DRAGON cluster-randomized controlled trial, which was designed to assess the effectiveness and cost-effectiveness of a childhood obesity prevention intervention in Guangzhou, China.^24,25^ Children had baseline measurements, as described below, in 2015 when they were 6 to 7 years old.

Details about eligibility and measurements are available elsewhere.^24,25^ In brief, 40 schools from among 353 eligible nonboarding, state-funded primary schools in Guangzhou, China, were selected using a random number generator and recruited. Informed consent was sought for each year-1 child (aged 6 to 7 years) in selected schools, from their parents or guardians. All outcomes were collected at the individual level by independent and trained assessors (research staff) using standardized trial procedures.

### Anthropometric Measurements

Height and weight measurements were undertaken of children without shoes and in light clothing. Standing height was measured at least twice with a TGZ-type height tester (Dalian). Weight was measured with an electronic scale (JH-1993T, Weighing Apparatus Co Ltd, Dalian, China). Body mass index (BMI) was calculated as weight in kilograms divided by the square of height in meters (kg/m^2^). The World Health Organization 2007 growth charts were used to calculate BMI *z* scores and to categorize the children into underweight, healthy weight, overweight, and obese weight groups.^26^

### Measurement of HRQOL

The Chinese version of the CHU-9D (CHU9D-CHN)^27^ and PedsQL™, which are both generic instruments, were used to measure HRQOL. Both instruments were researcher-administered considering the young age of the participants.

Ideally, utility-based HRQOL in children should be measured using an instrument specifically designed for them.^28^ Although there is no gold standard for measuring utility-based HRQOL in primary school–age children, previous research has shown that the CHU-9D is an appropriate choice.^29^ It is a preference-based instrument, not specific to any one condition or disease, and designed for application in economic evaluation of prevention, treatment, and service programs targeted at young people where the QALY is the desired outcome measure.^30^ The CHU-9D-CHN instrument combines 9 dimensions of HRQOL: worried, sad, pain, tired, annoyed, schoolwork/homework, sleep, daily routine, and ability to join in activities^31,32^ (**Appendix 1**). Each dimension comprises 5 severity levels, resulting in 1 953 125 unique health states associated with the measure. Individual responses from the questionnaires were transformed into utility weights derived from a UK general population sample using an algorithm developed by Stevens et al.^31,32^ This presents a possible utility value set of between 0.33 (worst health state) and 1 (best health state). The CHU-9D-CHN instrument has a Chinese tariff set available for estimating utility values, but according to the instrument developers (Gang Chen and Julie Ratcliffe, personal email communication), these Chinese-specific preference weights were still in development and required further validation at the time of the study. Therefore, the UK tariff set was used for the main analysis, and the Chinese tariff set was applied as part of an exploratory analysis.^33^

The PedsQL™ is a widely used HRQOL instrument validated for use with young children over 5 years old in diverse populations.^34,35^ It has good reliability and validity in both sick and healthy populations.^34,35^ The PedsQL™ is a non-preference-based instrument which does not apply any explicit weighting between item domains and therefore cannot be used to generate utility values for the construction of QALYs. However, it would be expected to produce HRQOL values that move in the same direction as the utility values and therefore was included as a reference to assess HRQOL and to assess movement against the utility values. The PedsQL™ is a 23-item instrument comprising 4 domains: physical (8 items), emotional (5 items), social (5 items), and school (5 items) functioning.^34^ Each item has 5 response options: never, hardly ever, sometimes, often, and almost always. Emerging from the instrument is a score (transformed onto a 0-100 scale) for each domain and a score for total HRQOL. Decreasing scores indicate poorer HRQOL. For this study the validated Chinese version of the PedsQL™ 4.0 instrument was used.^36^ The mean score for each of the 4 domains was calculated by summing the values for the relevant items and dividing by the number of items answered. This process generated a mean for the total score (mean of all items), for the physical health score (mean of physical functioning items) and for the psychosocial health score (mean of emotional, social and school functioning items).

### Demographic Measurements

Data on participants’ date of birth and gender were obtained from school records. Parental education level was collected through a parent-completed questionnaire, coded as a binary variable (did or did not attend university).

### Relationship Between Weight Status and HRQOL

The relationship between HRQOL and weight status category (defined as either “overweight/obese vs healthy/ underweight” or “underweight vs healthy weight, overweight, or obese”) was examined using descriptive analyses. A linear mixed regression model (with random effect for school), adjusted for potential confounders (age, gender, and parents’ education) was used to compare the CHU-9D utility values (using the UK and Chinese tariffs) between 2 weight status groups (overweight/obese compared with healthy/underweight). Separate analyses or models were used to assess whether any relationship differed in boys compared with girls with the prior hypothesis that being overweight would negatively impact HRQOL in girls more than in boys.

Differences in HRQOL scores between groups were estimated using the nonparametric test for trend (across ordered categories of a variable). All statistical analyses were undertaken in 2019, using Stata version 13 (StataCorp).

## RESULTS

### Participant Characteristics

Complete baseline data (including PedsQL™ total score and its subscales, CHU-9D-CHN dimensions and utility value, height and weight (converted to BMI *z* score and weight status), gender, age, and parents’ education level) were available for 1539 out of 1640 children (93.8% of those who consented and participated in study measurements) and are described in Table 1.

**Table 1.** Characteristics of the Study Population

Gender, n (%)	
Male	831 (54.0)
Female	708 (46.0)
Age (years), mean (SD)	6.6 (0.42)
Measures of socioeconomic status	
Maternal university education: n (%)	
Yes	963 (62.6)
No	576 (37.4)
Paternal university education: n (%)	
Yes	1005 (65.3)
No	534 (34.7)
Weight status, n (%)	
Underweight	75 (4.9)
Healthy weight	1189 (77.2)
Overweight	165 (10.7)
Obese	110 (7.2)
Underweight/healthy weight compared with overweight/obese, n (%)	
Underweight/healthy weight	1264 (82.1)
Overweight/obese	275 (17.9)
BMI, mean (SD)	15.45 (2.13)
BMI *z* score, mean (SD)	-0.12 (1.29)
CHU-9D mean score (SD)	
Using UK tariff	0.937 (0.068)
Using Chinese tariff	0.920 (0.094)
PedsQL™ mean score (SD)	
Total scale score	82.92 (11.21)
Physical functioning	83.67 (13.15)
Psychosocial functioning	82.52 (12.36)
Emotional functioning	81.69 (17.54)
Social functioning	84.09 (15.30)
School functioning	81.77 (15.36)

The mean age of the children was 6.6 years (SD, 0.42); 54% were male. Around two-thirds of parents were educated to university degree level or above. The mean BMI *z* score was -0.12 (SD, 1.29), while more than 17% of the children lived with overweight (10.7%) or obesity (7.2%); this is comparable to national data from China for overweight and obesity in the same age group (20.4%).^21^ The mean utility scores of the total sample was, on average, slightly higher for CHU-9D-CHN using the UK tariff (mean, 0.937 [SD, 0.068]) compared with using the Chinese tariff (mean, 0.920 [SD, 0.094]). The mean total PedsQL™ score was 82.92 (SD, 11.21).

### Relationship Between Weight Status and HRQOL

Table 2 summarizes the CHU-9D utility values and PedsQL™ total scores according to the weight status of the children. The direction of the relationships were similar between the instruments. The mean and median utility scores using both UK and Chinese tariffs and mean and median PedsQL™ total scores were all marginally higher for children who were overweight or obese compared with those who were not, but the differences were not statistically significant.

**Table 2.** Mean (SD) and Median (IQR) for CHU-9D and PedsQL™ Scores Based on Weight Status

	**No. (%)**	**Mean (SD), Median (IQR)**		
		**CHU-9D Utility, UK Tariff**	**CHU-9D Utility, Chinese Tariff**	**PedsQL**™ **Total Score**
Weight status group				
Underweight	75 (4.9)	0.942 (0.067), 0.963 (0.908-1.000)	0.923 (0.092), 0.938 (0.873-1.000)	82.47 (12.06), 85.86 (72.82-92.39)
Healthy weight	1189 (77.2)	0.936 (0.069), 0.962 (0.900-1.000)	0.919 (0.095), 0.943 (0.876-1.000)	82.84 (11.13), 83.69 (76.08-91.30)
Overweight	165 (10.7)	0.941 (0.064), 0.963 (0.909-1.000)	0.925 (0.086), 0.955 (0.874-1.000)	83.18 (11.65), 85.86 (76.08-91.30)
Obese	110 (7.2)	0.939 (0.071), 0.962 (0.914-1.000)	0.921 (0.), 0.943 (0.890-1.000)	83.69 (10.94), 86.95 (77.17-91.30)
*P* value^a^		0.73	0.89	0.29
Weight status group				
Underweight/healthy weight	1264 (82.1)	0.936 (0.069), 0.963 (0.901-1.000)	0.919 (0.095), 0.943 (0.875-1.000)	82.82 (11.18), 83.69 (76.08-91.30)
Overweight/obese	275 (17.9)	0.940 (0.067), 0.964 (0.909-1.000)	0.923 (0.090), 0.944 (0.876-1.000)	83.38 (11.35), 85.86 (76.08-91.30)
*P* value^a^		0.38	0.66	0.27

<sup>a</sup> Nonparametric test for trend.

Table 3 shows the results of the linear mixed regression model which compared the CHU-9D utility score between the 2 weight status groups, adjusted for potential confounders (age, gender, and parents’ education). The results were similar to the unadjusted analyses with marginally higher, but statistically nonsignificant CHU-9D utility values for children with overweight or obesity, compared with those with underweight or of healthy weight. Girls had slightly higher mean CHU-9D utility values compared with boys (*P*=0.001 and *P*=0.003 for UK and Chinese tariffs respectively), while children whose parents had a university education reported a lower HRQOL (not statistically significant).

**Table 3.** Linear Mixed Regression Model to Explore Association Between Weight Category and HRQOL^a^

**Variables**	**CHU-9D Utility Score: UK Tariff**			**CHU-9D Utility Score: Chinese Tariff**		
	**Mean Difference**	**95% CI**	***P* Value**	**Mean Difference**	**95% CI**	***P* Value**
Age (months)	0.001	(0.000, 0.001)	**0.01^b^**	0.001	(0.000, 0.002)	**0.01^b^**
Weight						
Underweight/healthy weight						
Overweight/obese	0.005	(-0.003, 0.014)	0.25	0.004	(-0.007, 0.016)	0.45
Gender						
Male	**—**					
Female	0.011	(0.005, 0.018)	**0.001^b^**	0.014	(0.004, 0.023)	**0.003^b^**
Mother’s university education						
No	**—**					
Yes	-0.002	(-0.012, 0.005)	0.46	-0.004	(-0.017, 0.007)	0.41
Father’s university education						
No	**—**					
Yes	-0.001	(-0.010, 0.007)	0.80	-0.002	(-0.009, 0.014)	0.66

^a^ Measured by CHU-9D, adjusting for potential confounders.

^b^ Significant at *P*=0.05.

When separate analyses/models were run for boys and girls (**Table 4** and **Table 5**), the findings were similar to those for the main analysis, with no statistically significant difference in HRQOL by weight status for either gender. In addition, the regression analysis was re-run with binary data: healthy weight against overweight/ obese (omitting the underweight category). As expected, the results were similar to the previous regression analysis: marginally higher, but statistically nonsignificant CHU-9D utility values for children with overweight/ obesity, compared with those with healthy weight.

**Table 4.** Mean (SD) and Median (IQR) for CHU-9D and PedsQL™ Scores Based on Weight Status by Gender

	n	**Boys**			n	**Girls**		
**CHU-9D Utility, UK Tariff**	**CHU-9D Utility, Chinese Tariff**	**PedsQL™ Total Score**	**CHU-9D Utility, UK Tariff**	**CHU-9D Utility, Chinese Tariff**	**PedsQL™ Total Score**			
Weight status group								
Underweight/ healthy weight								
Mean (SD)	641	0.931 (0.072)	0.913 (0.099)	82.10 (11.74)	623	0.942 (0.065)	0.926 (0.091)	83.56 (10.54)
Median (IQR)		0.951 (0.897-1.000)	0.938 (0.874-1.000)	83.69 (75.00-91.30)		0.963 (0.904-1.000)	0.955 (0.880-1.000)	84.78 (77.17-91.30)
Overweight/obese								
Mean (SD)	190	0.936 (0.072)	0.918 (0.096)	82.93 (11.68)	85	0.951 (0.051)	0.935 (0.072)	84.41 (10.58)
Median (IQR)		0.963 (0.903-1.000)	0.943 (0.872-1.000)	85.86 (76.08-91.30)		0.963 (0.914-1.000)	0.955 (0.891-1.000)	86.95 (79.34-91.30)
***P* value^a^**		0.29	0.41	0.28		0.45	0.79	0.38
Weight status group								
Underweight								
Mean (SD)	35	0.923 (0.070)	0.899 (0.096)	79.93 (14.55)	40	0.959 (0.060)	0.944 (0.084)	84.70 (8.97)
Median (IQR)		0.929 (0.877-1.000)	0.922 (0.815-1.000)	83.69 (69.56-92.39)		0.989 (0.924-1.000)	0.998 (0.913-1.000)	86.95 (76.08-92.39)
Healthy weight								
Mean (SD)	606	0.932 (0.072)	0.914 (0.099)	82.23 (11.56)	583	0.940 (0.065)	0.925 (0.091)	83.48 (10.64)
Median (IQR)		0.951 (0.900-1.000)	0.939 (0.876-1.000)	83.69 (75.00-91.30)		0.963 (0.902-1.000)	0.953 (0.875-1.000)	84.78 (77.17-91.30)
Overweight								
Mean (SD)	108	0.937 (0.069)	0.918 (0.094)	82.56 (12.26)	57	0.950 (0.052)	0.939 (0.065)	84.35 (10.38)
Median (IQR)		0.963 (0.893-1.000)	0.953 (0.860-1.000)	85.86 (76.08-91.30)		0.963 (0.914-1.000)	0.955 (0.896-1.000)	85.86 (79.34-91.30)
Obese								
Mean (SD)	82	0.934 (0.077)	0.918 (0.100)	83.41 (10.92)	28	0.953 (0.051)	0.928 (0.086)	84.53 (11.15)
Median (IQR)		0.951 (0.914-1.000)	0.943 (0.890-1.000)	86.41 (76.08-91.30)		0.963 (0.916-1.000)	0.940 (0.883-1.000)	87.77 (80.97-91.30)
***P* value^a^**		0.27	0.29	0.22		0.84	0.58	0.57

^a^ Nonparametric test for trend.

**Table 5.** Linear Mixed Regression Model Exploring Association Between Weight Category and HRQOL by Gender^a^

	**CHU-9D Utility Score**											
	**Boys**						**Girls**					
	**UK Tariff**			**Chinese Tariff**			**UK Tariff**			**Chinese Tariff**		
	**Mean Diff**	**95% CI**	***P* Value**	**Mean Diff**	**95% CI**	***P* Value**	**Mean Diff**	**95% CI**	***P* Value**	**Mean Diff**	**95% CI**	***P* Value**
Age (months)	0.001	0.000, 0.002	**0.03^b^**	0.001	0.000, 0.003	**0.02^b^**	0.001	0.000, 0.002	**0.02^b^**	0.001	0.000, 0.003	**0.01^b^**
Weight												
Underweight/ healthy weight												
Overweight/obese	-0.004	-0.007, 0.016	0.44	0.003	-0.011, 0.021	0.56	0.008	-0.007, 0.021	0.31	0.007	-0.013, 0.027	0.49
Mother’s university education												
No	**—**											
Yes	-0.001	-0.021, 0.013	0.97	-0.003	**-**0.014, 0.021	0.72	-0.001	-0.011, 0.013	0.98	-0.002	-0.019, 0.014	0.75
Father’s university education												
No												
Yes	-0.003	-0.017, 0.016	0.54	-0.004	**-**0.021, 0.015	0.71	-0.002	-0.014, 0.012	0.77	-0.004	-0.012, 0.022	0.62

Abbreviation: Mean Diff, mean difference.

^a^ Measured by CHU-9D, adjusting for potential confounders.

^b^ Significant at *P*=0.05.

## DISCUSSION

### Principal Findings

The findings suggest that although the HRQOL in this study population was marginally higher in children who lived with overweight or obesity compared with children of healthy weight, these differences were not statistically significant. The findings were the same for subgroup analysis based on gender.

### Strengths and Limitations

Strengths include the large sample size (1539 children), diverse population (selected to include a range of socioeconomic backgrounds), and standardized data collection procedures as part of the randomized controlled trial. The CHIRPY DRAGON study benefited from a partnership with the Chinese local health authority, which permitted a random selection of 40 primary schools from all eligible schools in Guangzhou city. According to the Chinese classification of socioeconomic status of urban districts for population health surveillance, the CHIRPY DRAGON sample was representative of the urban population of 6- to 7-year-old children in China. Furthermore, this study is one of the very few studies worldwide and the first study in China that collected utility-based HRQOL information in children as young as 6 years. It used both UK and Chinese tariffs for calculating the utility scores and reports the results of CHU-9D in direct comparison with the established, validated PedsQL™ instrument.

The study had some limitations. In this study, the “underweight” and “healthy weight” children were pooled into one weight status category. Although some studies have reported on the HRQOL of underweight children in comparison with those of healthy weight,^37^ this could not be explored in this study as the underweight sample size was too small (5% of the total). Further limitations relate to the way HRQOL information was collected from children. There might have been an influence on how children completed the questionnaire as all items and possible responses within the CHU-9D-CHN and the PedsQL™ were read aloud to children, on a one-to-one basis, by the research staff. This decision was made as children as young as 6 years have varying reading abilities which makes self-completion problematic. Analysis was limited to the data collected as part of the trial; and data related to other potential factors that could have influenced HRQOL in this age group, such as existence of support networks and friendship groups, were not collected. Furthermore, the analysis was cross-sectional, using the trial baseline data collected at one time point, and therefore did not account for any fluctuations in weight status due to variable rates of growth in children over time.

### Comparison With Other Studies

There exists no robust evidence on the direction of relationship between weight status and HRQOL in a childhood population. This is compounded by the challenging nature of measuring HRQOL in a pediatric population more generally.^38^ In 4 previous studies that have explored this relationship (3 UK-based studies using the CHU-9D^15–17^ and 1 US-based study using the Health Utilities Index [HUI®] instrument^18^), the direction of effect was opposite to that found in this study (lower HRQOL in participants with overweight or obesity compared with their underweight or healthy-weight counterparts). However, like this study, the differences were not statistically significant (no evidence of a negative relationship between health utility and weight status in children aged 5-6 years,^15^ 6-7 years,^16^ or 5-10 years,^17^ or children and adolescents aged 5-18 years,^18^ was found). In contrast, however, the findings of one recent study from Australia using the CHU-9D in children aged 9-12 years^19^ and one study from the UK using the EQ-5D-Y in children aged 11-15 years^20^ found a significant negative relationship between weight and HRQOL. Overall, the relationship between weight status and HRQOL appears to be affected by age and contextual factors related to the culture and setting.

The weak relationship between weight status and utility-based HRQOL may be attributed to the CHU-9D not being sensitive enough to detect a difference in very young children as it was originally developed for use with children aged 7-11 years.^39^ Although the findings of a UK-based study suggested the instrument to be acceptable and feasible to administer for children aged 6-7 years,^29^ there are still concerns with regard to the instrument’s reliability in young children.^29,40^

A wide range of previous studies demonstrate that childhood obesity is associated with lower HRQOL when non-utility instruments are used.^4–8,20,22,23^ However, the findings are not consistent, particularly in younger children. For example, in addition to this study, another study from China^21^ and one from the UK^15^ in children of similar age found no significant relationship between weight status and HRQOL measured using the PedsQL™.

Cultural differences may play a role. Many Chinese parents and particularly grandparents aspire for children to be overweight, as this is taken to be a sign of health, growth, and prosperity.^12,41^ Obesity trends follow a different pattern in China compared with high-income countries.^13,14^ Because of these different cultural values, weight stigma, which can lead to discrimination toward children with obesity, might not be as prevalent within a Chinese setting. The lack of association between weight and HRQOL may also be related to the fact that comorbidities attached to obesity do not substantially affect utility in this age group. Possibly, it is only once these children approach adolescence that the effects of obesity starts to impact negatively on HRQOL.^5^

### Implications for Practice, Policy, and Research

The results of this study have potential methodological and policy implications in terms of how the cost-effectiveness of population-based childhood obesity interventions are measured. Obesity prevention and treatment interventions tend to target young populations; therefore, information about how weight status is associated with HRQOL in utility terms in this age group is crucial for the design of economic evaluations. Within health economic studies conducted globally, utility values are often used to derive QALYs to inform resource allocation decisions. To help inform the methods of economic evaluations alongside clinical trials of childhood obesity prevention and treatment interventions, future studies need to determine the relationship between weight status and utility-based HRQOL in different age groups, and across different country settings. In addition, it is recommended that future studies aiming to prevent obesity in young children (age 6-12) do not rely solely on HRQOL measures for economic evaluation, and capture clinical or well-being outcomes as well.

## CONCLUSIONS

The results of this study suggest that HRQOL measured using both the CHU-9D and the PedsQL™ is slightly (nonsignificantly) higher among Chinese children who live with overweight or obesity compared with those with underweight or of healthy weight. Findings of the gender subgroup analysis were consistent with the main analysis. Some studies from high-income countries suggest that overweight and obesity in children is negatively associated with HRQOL. However, the extent of the relationship, how it varies across age groups, and how this translates to utility-based HRQOL across different settings is as yet under-researched.

### Author Contributions

MZ, PA, BL, and EF contributed to the design of this study. BL, PA, MP, and KKC conceived the main CHIRPY DRAGON study and with EF and JM designed the trial, on which the analysis was based. Data acquisition for the trial was overseen by BL and LWJ, with support from RL and WL. MZ analyzed the data and drafted the manuscript. PA, BL, and EF oversaw and shaped the analysis. All authors commented on and approved the final manuscript.

### Consent

Full ethics approvals were obtained from the Life and Health Sciences Ethical Review Committee at the University of Birmingham (March 2, 2015) and the Ethical Committee of Guangzhou Centre for Disease Control and Prevention (December 1, 2014). Informed consent was sought for each child participant from their parents/guardians.

## Supplementary Material

Supplementary Online Material
