# Supplementary material for: Relationship Between Weight Status and Health-Related Quality of Life in School-age Children in China
Source: J Health Econ Outcomes Res. 2022 Mar 7;9(1):75–81. doi: 10.36469/jheor.2022.32414 (PMC8901243; doi:10.36469/jheor.2022.32414)
Supplement: Supplementary Online Material [file jheor_2022_9_1_32414_83791.pdf]

Journal of Health Economics and Outcomes Research

Online Supplementary Material

Relationship Between Weight Status and Health-Related Quality of Life in School-age Children in China. *JHEOR*. **2022;9(1):75-81**. doi:10.36469/jheor.2022.32414

Appendix 1: CHU-9D-CHN Instrument

This supplementary material has been provided by the authors to give readers additional information about their work.

This is an open-access article distributed under the terms of the Creative Commons Attribution 4.0 International License (CCBY-4.0). View this license's legal deed at http://creativecommons.org/licenses/by/4.0 and legal code at http://creativecommons.org/licenses/by/4.0/legalcode for more information.

**Appendix 1: CHU-9D-CHN Instrument**
**Thinking about today…**
**A1. Worried**
☐ I don't feel worried today.
☐ I feel a little bit worried today.
☐ I feel a bit worried today.
☐ I feel quite worried today.
☐ I feel very worried today.
**A2. Sad**
☐ I don't feel sad today.
☐ I feel a little bit sad today.
☐ I feel a bit sad today.
☐ I feel quite sad today.
☐ I feel very sad today.
**A3. Pain**
☐ I don't have any pain today.
☐ I have a little bit of pain today.
☐ I have a bit of pain today.
☐ I have quite a lot of pain today.
☐ I have a lot of pain today.
**A4. Tired**
☐ I don't feel tired today.
☐ I feel a little bit tired today.
☐ I feel a bit tired today.
☐ I feel quite tired today.
☐ I feel very tired today.
**A5. Annoyed**
☐ I don't feel annoyed today.
☐ I feel a little bit annoyed today.
☐ I feel a bit annoyed today.
☐ I feel quite annoyed today.
☐ I feel very annoyed today.
**A6. Schoolwork/Homework (such as reading, writing, doing lessons)**
☐ I have no problems with my schoolwork/homework today.
☐ I have a few problems with my schoolwork/homework today.
☐ I have some problems with my schoolwork/homework today.
☐ I have many problems with my schoolwork/homework today.
☐ I can't do my schoolwork/homework today.
**A7. Sleep**
☐ Last night I had no problems sleeping
☐ Last night I had a few problems sleeping
☐ Last night I had some problems sleeping
☐ Last night I had many problems sleeping
☐ Last night I couldn't sleep at all

**A8. Daily routine (things like eating, having a bath/shower, getting dressed)**
☐ I have no problems with my daily routine today.
☐ I have a few problems with my daily routine today.
☐ I have some problems with my daily routine today.
☐ I have many problems with my daily routine today.
☐ I can’t do my daily routine today.
**A9. Able to join in activities (things like playing out with your friends, doing sports, joining in things)**
☐ I can join in with any activities today.
☐ I can join in with most activities today.
☐ I can join in with some activities today.
☐ I can join in with a few activities today.
☐ I can join in with no activities today.
